# Severe Vitamin D Deficiency Is Associated With Increased Expression of Inflammatory Cytokines in Painful Diabetic Peripheral Neuropathy

**DOI:** 10.3389/fnut.2021.612068

**Published:** 2021-03-10

**Authors:** Gong Xiaohua, Luo Dongdong, Niu Xiaoting, Chen Shuoping, Shen Feixia, Yang Huajun, Zhou Qi, Chen Zimiao

**Affiliations:** ^1^Department of Endocrinology and Metabolism, The First Affiliated Hospital of Wenzhou Medical University, Wenzhou, China; ^2^Department of Endocrinology and Metabolism, The Second Affiliated Hospital of Dalian Medical University, Dalian Medical University, Dalian, China; ^3^Department of Neurology, The First Affiliated Hospital of Wenzhou Medical University, Wenzhou, China; ^4^Songqiao Hospital of Wanquan Town, Wenzhou, China

**Keywords:** type 2 diabetes mellitus, severe vitamin D deficiency, inflammatory cytokine, painful diabetic peripheral neuropathy, IL-6, TNF–α

## Abstract

**Background:** The exact pathogenic mechanism of the painful diabetic peripheral neuropathy (DPN) is poorly understood. Our study aimed to evaluate the association amongst vitamin D status, inflammatory cytokines, and painful DPN.

**Methods:** A total of 483 patients were divided into three groups, i.e., diabetes without DPN (no-DPN, *n* = 86), diabetes with painless DPN (painless DPN, *n* = 176) and diabetes with painful DPN (painful DPN, *n* = 221) groups. Basic information and laboratory results were collected. The concentrations of vitamin D (25-hydroxyvitamin D), high-sensitivity C-reactive protein, interleukin (IL)-2, IL-4, IL-6, IL-10, tumor necrosis factor-α (TNF-α), and interferon-γ (IFN-γ) were also measured.

**Results:** The prevalence of severe vitamin D deficiency (<10 ng/mL) was more common in the painful DPN group than in the painless DPN and no-DPN groups (25.8,12.5, and 8.1%, respectively, *P* < 0.01). Cases in the painful DPN group had significantly higher concentrations of IL-6 (*P* < 0.01) and TNF-α (*P* < 0.01) than those in the two other groups. The multivariate logistic analysis showed that severe vitamin D deficiency, IL-6, and TNF-α were independent risks for painful DPN after adjusting for confounding factors. Furthermore, the vitamin D status had significantly negative correlations with IL-6 (*r* = −0.56, *P* < 0.01) and TNF-α (*r* = −0.47, *P* < 0.01) levels.

**Conclusion:** Severe vitamin D deficiency was an independent risk factor for the painful DPN. Severe vitamin D deficiency status may play a role in the painful DPN pathogenesis through elevated IL-6 and TNF-α levels.

## Introduction

China has the world's largest diabetes epidemic, which continues to increase. In China, the latest publication has presented that the prevalence of diabetes has increased to 12.8% nationwide (1). A total of 693 million people are estimated to have diabetes by 2045 (2). An estimated 60–70% of patients with diabetes mellitus (DM) suffer from neuropathies, and the diabetic peripheral neuropathy (DPN) is common amongst patients with diabetes (3). About 25% of people with diabetic neuropathy also develop painful DPN (4, 5). Major neuropathic symptoms include burning, aching and electric shock-like pains, resulting in a huge burden on patients' health and the society (6, 7). Painful DPN is a remarkable economic burden for patients, families and the society (5, 7). Despite recent advances, the pathogenesis of painful DPN remains incomplete. Previous studies indicate an association between vitamin D deficiency and the painful DPN. Mohammad et al. have reported that the vitamin D status (deficient, insufficient, and sufficient vitamin D) and the neuropathic pain in patients with DM have no correlation (8). Shillo et al. have demonstrated that after adjustment for age, weight, activity score and sunlight exposure, people with painful DPN have significantly lower serum vitamin D level compared with those with painless DPN and DM without DPN (9). A recent study has suggested that vitamin D deficiency is related to painful DPN (10). Furthermore, Basit et al. have conducted a clinical trial to assess the effects of a single intramuscular dose of 600,000 IU vitamin D_3_ on the symptoms of painful DPN (11). Results show that vitamin D is an efficacious treatment for DPN. Another study also shows that the visual analog scale in patients with painful DPN after treatment with 2000 IU vitamin D_3_ decreases to 50%. A recent review is performed to investigate the effects of vitamin D supplementation on the signs and symptoms of DPN (12). Results show that vitamin D supplementation can improve subjective and objective pain scores in painful DPN. However, the mechanisms underlying the effect remain unclear.

Vitamin D is believed to maintain a balance between inflammation and immunosuppression (13). Observational studies show that elevated inflammation in chronic conditions, including osteoarthritis (14), hypertensive disorders in pregnancy (15), and diabetic foot infection (16), is linked to vitamin D deficiency. An animal experiment also shows that the increased levels of IL-1β and TNF-α are related to painful DPN in rats (17). Yanik's et al. findings suggest that IL-10 can reduce the pain behavior in an animal model of painful DPN (18).

The present study is based on the hypothesis that the vitamin D status plays a role in the painful DPN pathogenesis through elevated inflammation. To test our hypothesis, we have measured the vitamin D status and the circulating levels of hs-CRP, IL-2, IL-4, IL-6, IL-10, TNF-α, and IFN-γ in patients with painful DPN and evaluated the relationship between vitamin D and the abovementioned cytokines.

## Methods

### Study Design and Participants

Between April 2018 and August 2019, patients with type 2 diabetes mellitus (T2DM) were recruited from the Department of Endocrinology at the Affiliated Hospital of Wenzhou Medical University. All participants were consecutive patients. T2DM was diagnosed using 75 g oral glucose tolerance tests in accordance with the American Diabetes Association's criteria. The exclusion criteria included type 1 diabetes, hepatic failure, chronic renal impairment, inflammatory diseases, malignancy, hyperparathyroidism, recent vitamin D supplementation, central nervous system diseases, and causes of polyneuropathy other than diabetes. All participants in our study were divided into three groups, i.e., diabetes without DPN (no-DPN, *n* = 86), diabetes with painless DPN (painless DPN, *n* = 176), and diabetes with painful DPN (painful DPN, *n* = 221) groups.

### Data Collection

A detailed clinical history, including age, gender, diabetes duration, smoking, hypertension, and antidiabetic medication usage, was obtained from participants' medical records and through self-reporting. The body mass index (BMI) was calculated using the formula: BMI = weight (kg)/height (m^2^).

Blood samples were collected from the antecubital vein in the overnight fasting state. Blood samples were used for estimating renal and liver functions, plasma glucose (by using standard enzymatic methods), glycated hemoglobin (HbA1c, by using high-performance liquid chromatography) and lipid profiles (by using standard enzymatic methods), including total cholesterol (TC), high-density lipoprotein cholesterol (HDL-C), low-density lipoprotein cholesterol (LDL-C) and triglycerides (TG). The early-morning spot urine specimen was collected using immunoturbidimetry and the standard enzymatic method (Wako, Osaka, Japan) to assess the urinary albumin-to-creatinine ratio (UACR).

### Assessment of DPN

DPN was assessed using the Michigan Neuropathy Screening Instrument (MNSI), a validated screening tool for DPN. The MNSI included two separate assessments: a 15-item self-administered questionnaire and a structured examination of feet (MNSIE) that scored for abnormalities of appearance, presence of ulcers, vibration perception and ankle reflexes. All MNSIEs in our study were performed by a trained professional to reduce interviewer variability. The threshold for DPN was established via previous validation studies in adults and used a score of more than 2 on the MNSIE out of a total score of 8. The details of the examination are described in the previous study (19, 20).

### Assessment of the Neuropathic Pain

The neuropathic pain was diagnosed when the DN4 questionnaire gave a value ≥4. The DN4 questionnaire is a patient-reported symptom-based 10-item approach consisting of sensory descriptors and signs related to the bedside examination. This questionnaire was developed and validated by the French Neuropathic Pain Group. The cutoff value of 4/10 represented the highest percentage of correctly diagnosing patients (86.0%) and had sensitivity and specificity of 82.9 and 89.9%, respectively (20).

### Measurement of the Serum Vitamin D

25-hydroxyvitamin D was measured through the electrochemiluminescence immunoassay by using the Roche Modular E170 Analyzer (Roche Diagnostics GmbH, Switzerland). The vitamin D status of participants were classified in accordance with the Endocrine Society's clinical practice guidelines (sufficient vitamin D level, >30 ng/mL; insufficient vitamin D level, 20–30 ng/mL; deficient vitamin D level, 10–19.9 ng/mL and severe vitamin D deficiency, <10 ng/mL) (21).

### Serum Cytokine Levels

Serum samples collected for detection of cytokine concentrations were stored at −70°C. The serum concentrations of IL-2, IL-4, IL-6, IL-10, TNF-α, and IFN-γ in the samples were evaluated using the Luminex xMAP (Ceger Biotechnology Co., Ltd., China) in accordance with the manufacturer's instructions. Serum levels were measured in pg/mL. The hs-CRP was measured using the highly sensitive nephelometric assay (Ceger Biotechnology Co., Ltd., China).

### Statistical Analysis

The SPSS 21.0 software (SPSS Inc., Chicago, IL) was used for statistical analysis. Data were presented as mean ± SD, percentages or median values with interquartile range. Differences between groups were tested using ANOVA. Count data were tested using χ^2^ tests. The Spearman correlations were used for the bivariate analysis of the association between vitamin D and clinical/biochemical parameters. The multivariate logistic analysis was performed using identified independent variables, and odds ratios (OR) between the comparison groups were obtained with 95% confidence interval (CI). All tests were two-sided, and *P* < 0.05 was considered statistically significant.

## Results

A total of 483 patients (age = 28–79 years) were recruited for our study. Table 1 shows the baseline characteristics. No significant difference in gender, age, BMI, the proportion of smokers and hypertension was found amongst the no-DPN, painless DPN and painful DPN groups (all *P* > 0.05). No significant difference in the levels of TC and HDL-C was observed amongst the three groups (all *P* > 0.05). The levels of UACR and TG and the diabetes duration in the painless and painful DPN groups were significantly higher than those in the no-DPN group (all *P* < 0.01). The levels of HbA1c and LDL-C in the painful DPN group were significantly higher compared with those in the no-DPN and the painless DPN groups (all *P* < 0.01).

**Table 1:** Demographic data and inter-group comparisons of clinical/biochemical parameters.

	**No-DPN (*n* = 86)**	**Painless-DPN (*n* = 176)**	**Painful-DPN (*n* = 221)**	***P*-value**
Gender				0.91
Male	49	101	135	
Female	37	75	86	
Age (years)	54.3 ± 12.9	54.5 ± 13.0	54.0 ± 13.0	0.99
Diabetes duration(years)	5.0 ± 1.8	7.2 ± 2.9^*^	7.3 ± 3.0^*^	<0.01
Smoking(yes/no)	31/55	78/99	92/126	0.41
BMI (kg/m^2^)	23.1 ± 2.9	24.8 ± 3.0	25.0 ± 3.2	0.56
Waist circumference, cm	89.1 ± 10.5	91.7 ± 10.3	92.3 ± 11.6	0.19
Hypertension(yes/no)	47/39	106/70	142/79	0.29
HbA1c (%)	8.76 ± 1.8	8.8 ± 1.6	9.3 ± 1.6^*^^#^	<0.01
TC (mmol/L)	5.6 ± 0.8	5.6 ± 0.9	5.7 ± 0.9	0.36
TG (mmol/L)	1.9 ± 0.9	2.2 ± 0.9^*^	2.4 ± 0.8^*^	<0.01
HDL-C (mmol/L)	1.1 ± 0.3	1.1 ± 0.3	1.1 ± 0.3	0.24
LDL-C (mmol/L)	2.9 ± 0.8	2.9 ± 0.9	3.3 ± 0.8^*^^#^	<0.01
UACR (mg/g)	77.2 ± 115.1	133.7 ± 151.0^*^	136.2 ± 153.9^*^	<0.01

* Indicates to P < 0.05 when compared with the no-DPN group.

# Indicates to P < 0.05 when compared with the painless-DPN group.

*DPN, diabetic peripheral neuropathy; ACR, albumin creatinine ratio*.

No significant difference in vitamin D level was found amongst the three groups (*P* > 0.05, Table 2). A significant difference in the prevalence of severe vitamin D deficiency was found amongst the three groups. The prevalence of severe vitamin D deficiency was detected in 8.1, 12.5, and 25.8% of no-DPN, painless DPN and painful DPN participants, respectively (*P* < 0.01). No significant difference was observed in IL-4, IL-10, and IFN-γ levels amongst the three groups. The painful DPN group had significantly higher IL-6 and TNF-α levels compared with the no-DPN and the painless DPN groups (all *P* < 0.01). The painless and the painful DPN groups had significantly higher serum hs-CRP and IL-2 levels compared with the no-DPN group (*P* = 0.03 and *P* < 0.01, respectively).

**Table 2:** Vitamin D level and serum cytokine in study subjects.

	**No-DPN (*n* = 86)**	**Painless-DPN (*n* = 176)**	**Painful-DPN (*n* = 221)**	***P-*value**
Vitamin D (ng/ml)	20.5 ± 8.9	19.2 ± 8.8	18.3 ± 8.9	0.13
≥30, *n* (%)	18 (20.9)	30 (17.0)	37 (16.7)	<0.01
20–29.9, *n* (%)	27 (31.4)	42 (23.9)	45 (20.4)	
10–19.9, *n* (%)	34 (39.5)	82 (46.6)	82 (37.1)	
<10, *n* (%)	7 (8.1)	22 (12.5)	57 (25.8)^*^^#^	
IL-2 (pg/mL)	0.21 (0.06, 0.34)	0.51 (0.10, 0.72)^*^	0.56 (0.27, 0.60)^*^	<0.01
IL-4 (pg/mL)	0.65 (0.10, 0.75)	0.92 (0.20, 1.55	0.93 (0.19, 1.58)	0.08
IL-6 (pg/mL)	5.55 (3.19, 7.09)	7.26 (4.31–9.00)^*^	9.24 (4.94, 11.59)^*^^#^	<0.01
IL-10 (pg/mL)	3.09 (1.42, 3.97)	3.60 (1.86, 4.15)	3.14 (1.63, 4.01)	0.23
TNF-α (pg/mL)	4.90 (2.59, 6.16)	5.65 (3.24, 7.15)	12.36 (7.94, 15.53)^*^^#^	<0.01
IFN-γ (pg/mL)	0.50 (0.10, 0.55)	0.48 (0.1, 0.51)	0.45 (0.10, 0.48)	0.35
hs-CRP (mg/L)	0.97 (0.56, 2.78)	1.32 (0.72,3.15)^*^	1.43 (0.76, 3.41)^*^	0.03

* Indicates to P < 0.05 when compared with the no-DPN group.

# Indicates to P < 0.05 when compared with the painless DPN group.

*DPN, diabetic peripheral neuropathy*.

The Spearman correlations between the vitamin D and clinical and biochemical parameters are shown in Table 3. The BMI (*r* = −0.27, *P* = 0.03), TG (*r* = −0.41, *P* = 0.01) and UACR (*r* = −0.40, *P* = 0.02) showed significantly inverse correlations with vitamin D. Vitamin D was not significantly correlated with waist circumference, diabetes duration, and HbA1c.

**Table 3:** Spearman correlations of serum vitamin D levels with clinical and biochemical parameters.

**Variable**	**25 (OH) vitamin D**	
	***r***	***P***
Age (years)	−0.071	0.12
Diabetes duration(years)	−0.19	0.09
BMI (kg/m^2^)	−0.27	0.03
Waist circumference, cm	−0.10	0.06
HbA1c (%)	−0.08	0.22
TC (mmol/L)	−0.07	0.12
TG (mmol/L)	−0.41	0.01
HDL-C (mmol/L)	0.05	0.24
LDL-C (mmol/L)	−0.08	0.08
UACR (mg/g)	−0.40	0.02

**Figure 1:**
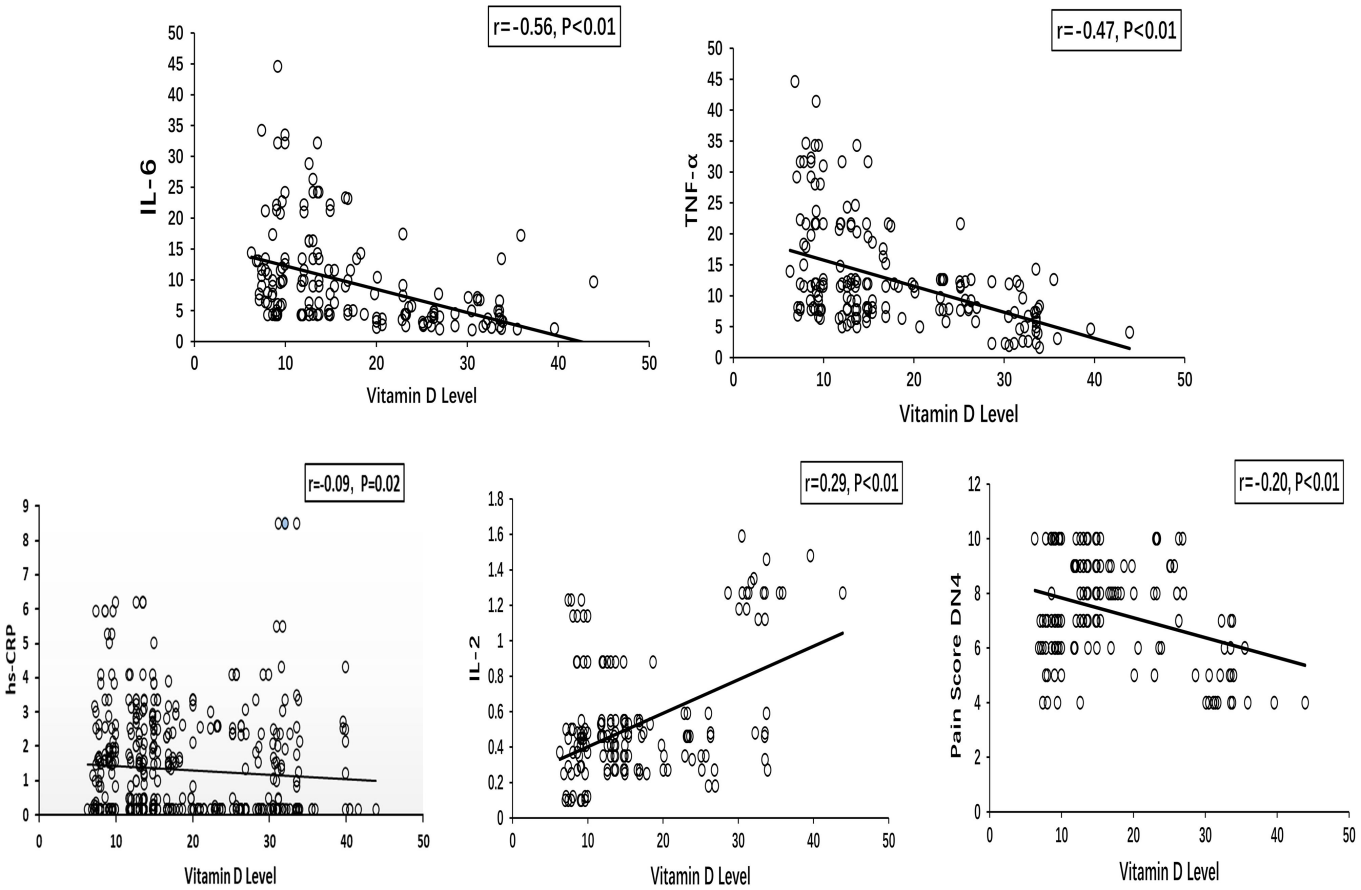
Spearman correlations between vitamin D level and inflammatory cytokines (IL-6, TNF-α, hs-CRP, and IL-2), pain score DN4 in painful DPN group. DPN, diabetic peripheral neuropathy.

The Spearman correlation analyses showed that the vitamin D level was negatively associated with IL-6 (*r* = −0.56, *P* < 0.01), TNF-α (*r* = −0.47, *P* < 0.01), IL-2 (*r* = 0.29, *P* < 0.01), and hs-CRP (*r* = −0.09, *P* = 0.02) levels (Figure 1). A negative correlation was identified between the vitamin D level and the pain score DN4 (*r* = −0.20, *P* < 0.01).

Multivariate logistic models were constructed to examine the risk factors for painful DPN. The univariable analysis showed that severe vitamin D deficiency, being male and HbA1c, TG, LDL-C, UACR, IL-6, and TNF-α levels were significantly associated with the risk of painful DPN.

**Figure 2:**
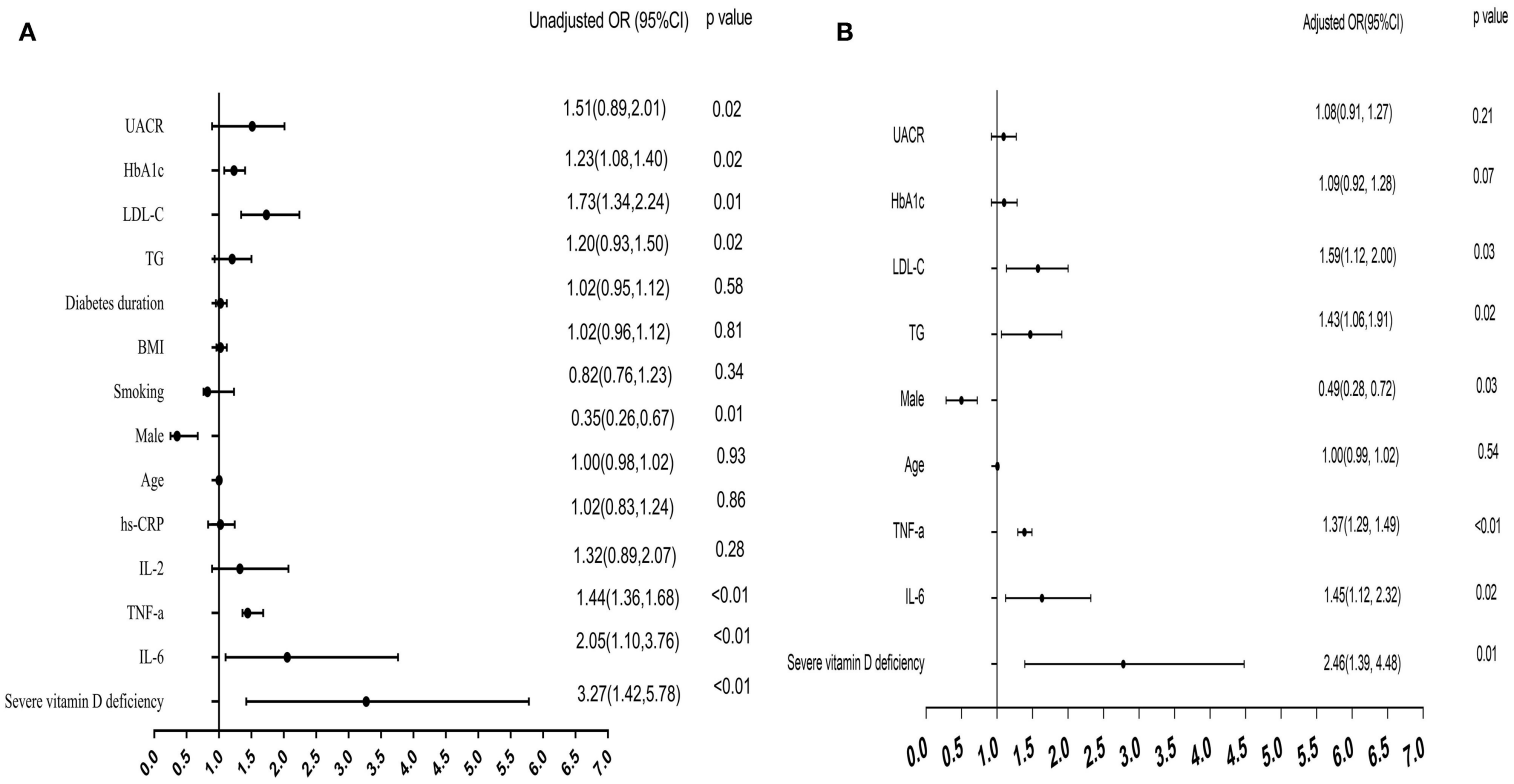
Forest plots for multivariate logistic analysis of variables for painful DPN. **(A)** Univariate logistic analysis: unadjusted odds ratio (OR) for each variable. **(B)** multivariate analysis: adjusted OR after adjustment for age, gender, HbA1c, TG, LDL-C, and UACR. DPN, diabetic peripheral neuropathy.

As shown in Figure 2, the multivariate analysis after adjustment for confounding factors, the severe vitamin D deficiency was still strongly associated with painful DPN (OR = 2.46, 95% CI, 1.39–4.48, *P* = 0.01). IL-6 (OR = 1.45, 95% CI, 1.12–2.32, *P* = 0.02) and TNF-α levels were also significantly associated with the presence of painful DPN after adjustment (OR = 1.37, 95% CI, 1.29–1.49, *P* < 0.01; Figure 2).

## Discussion

The 2001–2004 National Health and Nutrition Examination Survey has shown that the vitamin D deficiency (<30 ng/ml) is associated with self-reported peripheral neuropathy symptoms in American adults with diabetes (22). He et al. have found that the vitamin D deficiency is evidently associated with DPN, and vitamin D level <16.01 ng/mL predicts more than two-fold risk of the presence of DPN (23). Stephanotis et al. have reported that patients with vitamin D deficiency (<20 ng/mL) have a higher risk of symptomatic DPN (OR = 2.04, 95% CI = 0.99–4.02) compared with patients with 25-OH-D level of 30–40 ng/mL (24). Our study shows no significant difference in the prevalence of vitamin D deficiency (10–19.9 ng/mL) amongst the three groups (*P* = 0.053). The present study reveals that 25.8% of patients with painful DPN have severe vitamin D deficiency, whereas 8.1 and 12.5% of patients in the DM and the painless DPN groups have severe vitamin D deficiency. Our findings demonstrate a higher frequency of severe vitamin D deficiency in the painful DPN group than in the DM and the painless DPN groups (*P* < 0.01).

A negative correlation is identified between the vitamin D level and the pain score DN4 (*r* = −0.20, *P* < 0.01). The vitamin D level is lowest in people with the highest reported pain score. After adjusting for age, sex, smoking, BMI, diabetes duration, LDL-C, HbA1c, and UACR, severe vitamin D deficiency is an independent risk factor for painful DPN. Our findings suggest that severe vitamin D deficiency may have an important role in the pathogenesis of painful DPN.

Additionally, our study shows that inflammatory cytokines, such as hs-CRP, IL-2, IL-4, IL-6, IL-10, TNF-α, and IFN-γ, participate in the development and the progression of painful DPN. Our results reveal that the levels of IL-6 and TNF-α in patients in the painful DPN group are significantly higher compared with those in the no-DPN and the painless DPN groups (all *P* < 0.01). In addition, after adjusting for age, sex, smoking, BMI and diabetes duration, the levels of LDL-C, HbA1c, UACR, IL-6, and TNF-α are independent risk factors for painful DPN. A report from the Cooperative Health Research in the Region of Augsburg (KORA) F4/FF4 cohort with a mean follow-up of 6.5 years shows that IL-6 (OR = 1.31,95% CI, 1.00–1.71) and TNF-α (OR = 1.31, 95% CI, 1.03–1.67) levels are related to the incident distal sensorimotor polyneuropathy after adjusting for known distal sensorimotor polyneuropathy risk factors (25). Rosane et al. have reported that TNF-α can lead to the apoptosis of Schwann cells with subsequent damage to the peripheral nerve (26). TNF-α also improves the channel function in sensory neurons and leads to peripheral nerve injury, thereby inducing pain (27). Nadi et al. have found that exercises can significantly reduce TNF-α and CRP levels and have a consequent improvement in imbalance, pain and tingling (28). Our findings are in accordance with some previous reports. Machiavelli et al. have found that the serum IL-6 level increases in more than 40% of patients with DM and that the elevated IL-6 level has a significantly negative correlation with small and large nerve fiber functions and neuropathic pain (29). Backryd et al. have also demonstrated that patients with neuropathic pain have a higher level of IL-6 (30). A study by Angst et al. has also been reported that increased IL-6 concentration was noted in the patients with painful DPN (31).Data also show that IL-6 is a proinflammatory cytokine that has a significant effect on glial cells and neurons and trigger the onset of DPN in motion (32). Ma et al. have performed SC144 to block the IL-6-mediated signal transduction and demonstrate the function of IL-6 in the mechanical hyperalgesia induced by DM. Their data show that the administration of SC144 can effectively alleviate the mechanical hyperalgesia in STZ animals compared with controls (33). A recent study has also found a significant decrease in the neuropathy severity, a decrease in the IL-6 level and an increase in the IL-10 level after treatment with cholecalciferol (40,000 IU/week) for 24 weeks in patients with T2DM and DPN (34).

Data from the present study confirm that the vitamin D metabolite has strong associations with IL-6 (*r* = −0.56, *P* < 0.01) and TNF-α (*r* = −0.47, *P* < 0.01). The significantly negative correlations of vitamin D with inflammatory cytokines are consistent with those observed in previous studies. Tiwari et al. have evaluated the serum vitamin D level in 112 patients with diabetic foot infection and in 109 patients with diabetes but without foot infection. Tiwari et al. have found a significantly positive correlation with severe vitamin deficiency and high cytokine levels in patients with diabetes especially those with foot infection. Laird et al. have reported that the concentrations of IL-6 and CRP in individuals with vitamin D deficiency (<10 ng/mL) are significantly higher compared with those with sufficient vitamin D status (> 30 ng/mL) after adjusting for age, sex and BMI (*P* < 0.05) (35). Several mechanisms may explain the relationship of inflammatory cytokines with the vitamin D status (13, 36, 37).Vitamin D modulates nuclear transcription factors implicated in the cytokine generation and action by interacting with vitamin D response elements in the promoter region of cytokine genes. Moreover, vitamin D can activate the nuclear factor-β and regulate encoding proinflammatory cytokines. Calcitriol regulates the macrophage production of inflammatory factors via the calcium-dependent mechanism.

Some limitations exist in the present study. First, this study is a single-center cross-sectional study with a small number of cases. Second, the vitamin D level is affected by various factors. Seasonal variations in sun exposure and dietary sources of vitamin D are not analyzed. Further long-term prospective cohort or interventional studies are needed to confirm the causality in painful DPN.

In conclusion, our data suggest that severe vitamin D deficiency may play an important role in the progression of painful DPN. Furthermore, our analyses show that severe vitamin D deficiency is associated with altered inflammatory cytokines in painful DPN. In conclusion, severe vitamin D deficiency plays a role in painful DPN pathogenesis through elevated inflammation IL-6 and TNF-α levels. However, future studies with large number of subjects are needed to confirm these findings.

## Standard Biosecurity and Institutional Safety Procedures

All the biosafety measurements have been adopted and the institutional safety procedures are adhered. The laboratory of our institution has biosafety level 2 (BSL-2) standard where all standards and protocols are adopted as per the guidelines of Clinical and Laboratory Standards Institute (CLSI).

## Data Availability Statement

The raw data supporting the conclusions of this article will be made available by the authors, without undue reservation.

## Ethics Statement

The studies involving human participants were reviewed and approved by Ethics Committee of the First Affiliated Hospital of Wenzhou Medical College. The patients/participants provided their written informed consent to participate in this study.

## Author Contributions

CZ and SF designed and conceived the research. CS, NX, and YH recruited participants and collected data. LD and GX analyzed the data and interpreted the results. ZQ and CZ drafted the manuscript. All authors contributed to the article and approved the submitted version.

## Conflict of Interest

The authors declare that the research was conducted in the absence of any commercial or financial relationships that could be construed as a potential conflict of interest.
